# Highly Endemic, Waterborne Toxoplasmosis in North Rio de Janeiro State, Brazil

**DOI:** 10.3201/eid0901.020160

**Published:** 2003-01

**Authors:** Lílian Maria Garcia Bahia-Oliveira, Jeffrey L. Jones, Juliana Azevedo-Silva, Cristiane C.F. Alves, Fernando Oréfice, David G. Addiss

**Affiliations:** * Universidade Estadual do Norte Fluminense, Campos dos Goytacazes, Rio de Janeiro, Brazil; † Centers for Disease Control and Prevention, Atlanta , Georgia, USA; ‡ Escola de Medicina, Universidade Federal de Minas Gerais, Belo Horizonte, Brazil

**Keywords:** Toxoplasma gondii, toxoplasmosis, water, prevalence, Brazil, research

## Abstract

In Campos dos Goytacazes, northern Rio de Janeiro state, Brazil, reports of uveitis consistent with toxoplasmosis led to a survey of the prevalence and risk factors for *Toxoplasma gondii* infection in 1997–1999. The survey population was selected randomly from schools, randomly chosen communities, and an army battalion. Serum samples from 1,436 persons were tested. With results adjusted for age, 84% of the population in the lower socioeconomic group was seropositive, compared with 62% and 23% of the middle and upper socioeconomic groups, respectively (p<0.001). When multivariate analysis was performed, drinking unfiltered water was found to increase the risk of seropositivity for the lower socioeconomic (odds ratio [OR]: 3.0, 95% confidence interval [CI] 1.3 to 6.9) and middle socioeconomic (OR: 1.7, 95% CI 1.2 to 2.3) populations. We also found a high *T. gondii* seroprevalence in this Brazilian community. Drinking unfiltered water increased the risk of *T. gondii* seropositivity, indicating the potential importance of oocyst transmission in water in this region.

Toxoplasmosis, a zoonotic protozoal disease caused by *Toxoplasma gondii,* is horizontally transmitted to humans by the accidental ingestion of oocysts in cat feces or by eating raw or undercooked meat containing cysts (*1*). The infection is prevalent throughout the world, affecting a large proportion of adults who usually have no symptoms. Vertical transmission of toxoplasmosis from an acutely infected pregnant woman can cause serious disease in the fetus (*2*). In immunocompromised persons, a previously acquired *T. gondii* infection can be reactivated and result in severe illness, including encephalitis (*3*). In some areas of Brazil, the serologic prevalence of *T. gondii* infection ranges from 50% to 80% of the adult population; the highest values are found in some northern and southern states (*4*). In 1987, a survey of public school students in the capital of Rio de Janeiro State (Rio de Janeiro City) showed that the prevalence increased with age, reaching 71% for persons 16–20 years old (*5*). However, in the rest of Rio de Janeiro State, little information exists on the epidemiology of toxoplasmosis.

Water has been identified as a source of *T. gondii* infection in outbreaks (*6*,*7*), but it has not been well studied as a risk factor in toxoplasmosis-endemic areas. We report the results of a seroprevalence survey in northern Rio de Janeiro State in which we assessed exposure to known sources of *T. gondii* infection, as well as exposure to various types of water.

## Methods

### Study Area

Campos dos Goytacazes (Campos), located north of Rio de Janeiro with a population of about 400,000 persons, is the third most economically important city in the state. The Paraiba do Sul River, which supplies approximately 4.8 million persons in Brazil, provides the city with water and divides it into two parts. For this study, Campos was further divided into four geographic regions, according to predominantly urban or suburban (regions 1 and 2) or rural characteristics (regions 3 and 4).

### Study Population

A preliminary serologic survey in 1997 showed that 61 (82%) of 74 persons living in an extremely impoverished region of Campos and 15 (55%) of 27 public school children had antibodies to *T. gondii*. In the subsequent1997–1999 study presented here, we divided the city’s population into three socioeconomic strata. The first strata included persons living in poor communities in urban, suburban, or rural areas where neither residential sewage facilities nor municipally treated water were often available; these persons were from households that received less than US$150 per month in income (lower socioeconomic population). Lower middle-class children attending public schools, adult staff of the schools, and soldiers belonging to the county army battalion and adult members of their families constituted population 2 (middle socioeconomic population), in which monthly household income ranged from US$150–$500. Upper and middle class children attending private schools and the schools’ staff constituted population 3 (upper socioeconomic population); their households received more than US$500/month. Only some households in the lower socioeconomic population received treated water, whereas all households in the middle and upper socioeconomic populations received treated water.

To ensure a geographically representative sample of persons from the lower socio-economic population, 3 of 15 poor areas of the city were randomly selected. These areas represented 6% of the total population of Campos de Goytacazes. Households within these communities could not be randomly selected because the areas did not have a systematic spatial organization (such as conventional streets, quarters, or blocks). Therefore, we employed a visual sampling scheme in an attempt to select houses throughout the communities. In rural areas, because the houses were distant from each other, we went to all houses and invited persons found at home to participate.

To obtain a sample of persons from the middle socioeconomic population, we selected all public schools with >300 students (54 [61%] of the 89 public schools in Campos). In each school, all members of each odd-numbered class and all adult staff members were eligible for testing. The middle socioeconomic population also included soldiers from the only army base in Campos, ranging in age from 18 to 30 years, and adult members of their families (spouses and parents); 95% of the soldiers participated in the survey, and 80% brought at least one adult family member.

To obtain representatives of the upper socioeconomic population, we randomly selected two of the six private schools, from which children were chosen by using the same sampling scheme as for the middle socioeconomic population. Participants or their guardians provided written consent. This study was approved by the Brazilian Ministry of Health Ethics Commission (FIOCRUZ) and exempted by the Centers for Disease Control and Prevention Institutional Review Board (for data analysis).

### Questionnaire

For the evaluation of risk factors, we used a standard questionnaire, adapted from an outbreak of toxoplasmosis in Vancouver (*7*), which addressed (in 171 questions) exposure to known sources of *T. gondii* infection, as well as exposure to various types of water. A partial list of questionnaire variables included the following: age; sex; birth location; occupation; area of the city; urbanicity; number of persons in family; cat ownership; feeding cats raw or undercooked meat; where cats are kept (inside or outside); handling stray cats; exposure to cat feces; exposure to dogs and other pets; participation in birthing of animals and type of animal; gardening; exposure to soil; hand washing after soil exposure; hand washing before meals; types and frequency of meat and poultry eaten (including pork, chicken, beef, wild game, lamb, goat, sausage, locally prepared meats, and others); eating undercooked meat (for each type of meat); ingestion of unpasteurized cow’s milk, butter, and cheese, unpasteurized goat’s milk and cheese, eggs and raw eggs, homemade ice cream, nonleafy and leafy vegetables, and fruits; location of meat purchases; eating food grown in own garden; frequency of eating at restaurants and other locations; type of water used at home (well, unfiltered, filtered, spring [bottled] water); frequency of drinking various types of beverages and type of water used to prepare beverages; drinking directly from a lake, river, or stream; and whether a home water filter was used and how often it was changed. Parents or guardians responded for children <6 years old.

### Municipal Water Distribution and Treatment

Campos has two municipal water treatment plants. Since 1930, plant 1 has processed surface water from the river and supplies the major part of region 1 and all of region 2. In the early 1990s, plant 2 was built to supply part of region 1 with water from wells (more than 100 m deep). The rural areas do not receive treated water.

Plant 1 has sand and charcoal filters and treats water by flocculation, decantation, filtration, and chloramination; plant 2 has only sand filters and employs flocculation, decantation, filtration, oxidation of iron, and chloramination. The Brazilian Ministry of Health requires that the turbidity of treated water be below 5 nephelometric turbidity units and recommends that municipal drinking water be filtered at home with commercially available filters or boiled. Because of these recommendations, water from the faucet was considered unfiltered.

### Serologic Tests

Serum samples were tested at Núcleo de Apoio Diagnostico from the Federal University of Minas Gerais for toxoplasmosis by using a commercial ultramicro enzyme-linked immunosorbent assay (UMELISA) with fluorometric detection for anti-*Toxoplasma* IgG (UMELISA *Toxoplasma,* La Habana, Cuba). The correlation between the UMELISA and the Sabin-Feldman dye test, as well as the quality control of the UMELISA’s system, were evaluated by blindly exchanging 20 collected serum samples with the Research Institute, Palo Alto Medical Foundation, Palo Alto, California, USA. Twenty-six samples were also sent to the World Health Organization’s International Collaboration Centre for Research and Reference for *Toxoplasma*, Statens Serum Institut, Copenhagen, Denmark. Results from samples with values lower than 9 IU and higher than 15 IU in UMELISA agreed 100% with results from the dye test, and 99.7% of the survey samples were in this range of detection. Serum titers found by UMELISA (IU) were highly correlated with those found by the dye test (both low and high).

### Statistical Analyses

For examination of risk factors for T. gondii seropositivity, age was categorized into the following 7 groups: 0–9, 10–14, 15–19, 20–29, 30–39, 40–49, >50 years, and socioeconomic status was categorized into three groups as described previously. The data were initially examined with univariate analysis. For multivariate analysis, first a Cochran-Mantel-Haenszel summary statistic, controlling for age and socioeconomic status, was calculated for each risk factor examined in the survey (this was done because age and socioeconomic status were strongly associated with seropositivity). Then, factors that were found by the Cochran-Mantel-Haenszel summary statistic to have a significant (p<0.05) association with T. gondii seropositivity were entered into backward elimination logistic models, along with standard demographic variables. Variables were allowed to remain in the models at a p<0.1 level. Persons with high serologic test IU values (top 25%) and, separately, children (ages 0–14 years) with high serologic titers, were examined with separate models. Because of the numerous variables entered into each logistic model, p<0.01 was used as the cut-off for statistical significance. To compare seroprevalence among the three socioeconomic groups, the seroprevalence values were age-adjusted by the direct method to the age distribution of the combined study population.

## Results

A total of 1,436 persons were enrolled in the study. Of 381 persons from the lower socioeconomic population, 316 (83%) agreed to participate; of 1,059 persons from the middle socioeconomic population, 819 (77%) agreed to participate; and of 372 persons from the upper socioeconomic population, 301 (81%) agreed to participate. The age-adjusted seroprevalence values were: 84%, 62%, and 23% for the lower, middle, and upper socioeconomic populations, respectively. We observed an increasing prevalence with age for all three population groups (Figure). However, the prevalence of *T. gondii* infection among persons in each age group <40 years old was significantly (p<0.05, chi square or Fisher exact test) lower for the upper socioeconomic population than for the middle and lower socioeconomic populations.

**Figure:**
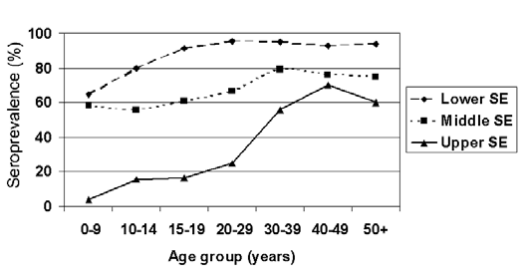
Serum prevalence by socioeconomic group and age for the lower, middle, and upper socioeconomic (SE) populations.

Age and socioeconomic status were the strongest predictors of *T. gondii* seropositivity. Table 1 shows the values for statistically significant variables (p<0.01) in the univariate analysis. All the variables assessing consumption of unfiltered or untreated water were significantly associated with *T. gondii* seropositivity with a 1.6- to 3-fold increased risk of infection. Having spring water in the house was protective (odds ratio [OR] 0.4 confidence interval [CI] 0.3 to 0.5). Increased risk for *T. gondii* seropositivity was also detected for owning a cat and eating unpasteurized butter. However, decreased risk for *T. gondii* seropositivity was detected for living in region 1, eating undercooked meat (general question about all types of meat only, not specific types of meat), eating kibbeh (ground meat with spices), eating food at home entertainment shows, and eating at least some of the time in restaurants. Soil contact was not associated with increased risk of infection, nor was infection associated with other types of meat, other pet exposure, hand washing, soil exposure, gardening, or eating fruits or vegetables.

**Table 1:** Univariate results showing *Toxoplasma gondii* seropositivity for statistically significant characteristics^a^

Characteristic	No. persons	% seropositive	Odds ratio	95% CI^c^
Age (yr)				
0–9	189	39.7	Ref.^b^	
10–14	585	45.5	1.3	0.9 to 1.8
15-19	256	63.7	**2.7**	**1.8 to 4.0**
20-29	161	70.8	**3.7**	**2.3 to 5.9**
30-39	91	83.5	**7.7**	**4.0 to 15.2**
40-49	89	83.2	**7.5**	**3.9 to 14.8**
50+	65	83.1	**7.5**	**3.5 to 16.2**
Socioeconomic population				
Lower	316	84.8	Ref.	
Middle	819	61.4	**0.3**	**0.2 to 0.4**
Upper	301	16.9	**0.04**	**0.02 to 0.06**
Location				
Urban	1040	49.9	Ref.	
Suburban	251	79.7	**3.9**	**2.8 to 5.6**
Rural	145	71.0	**2.5**	**1.7 to 3.7**
Regions 2-4	597	73.2	Ref.	
Region 1	839	45.9	**0.3**	**0.2 to 0.4**
Well water in house				
No	1159	54.0	Ref	
Yes	277	70.8	**2.1**	**1.6 to 2.7**
Unfiltered water in house				
No	1050	50.1	Ref.	
Yes	386	74.4	**2.8**	**2.2 to 3.6**
Spring water in house				
No	874	65.6	Ref.	
Yes	562	44.3	**0.4**	**0.3 to 0.5**
Drink at least some unfiltered water				
No	907	48.0	Ref.	
Yes	529	73.2	**3.0**	**2.4 to 3.7**
Drink at least some beverages made with unfiltered water				
No	968	49.5	Ref.	
Yes	468	73.3	**2.8**	**2.2 to 3.5**
Drink water directly from lakes, rivers or streams				
No	1160	55.2	Ref.	
Yes	276	65.9	**1.6**	**1.2 to 2.1**
Drink water directly from faucet				
No	699	46.2	Ref.	
Yes	737	67.7	**2.4**	**2.0 to 3.0**
Drank beverages made with well water in the past 12 months				
No	1173	54.7	Ref.	
Yes	263	68.4	**1.8**	**1.4 to 2.4**
Food Exposure:				
Ate undercooked meat in past 12 months				
No	877	59.9	Ref.	
Yes	559	53.1	**0.8**	**0.6 to 0.9**
Ate kibbeh the past 12 months				
No	1181	59.8	Ref.	
Yes	255	45.5	**0.6**	**0.4 to 0.7**
Ate unpasteurized butter in past 12 months				
No	983	54.5	Ref.	
Yes	453	63.1	**1.4**	**1.1 to 1.8**
Owned cat in past 12 months				
No	942	52.0	Ref.	
Yes	494	67.2	**1.9**	**1.5 to 2.4**
Ate food at home entertainment shows in the past 12 months				
No	923	62.6	Ref.	
Yes	513	47.6	**0.5**	**0.4 to 0.7**
Ate at least some of the time in restaurants in the past 12 months				
No	517	73.5	Ref.	
Yes	919	48.1	**0.3**	**0.3 to 0.4**

^a^p<0.01, in bold.

^b^ Ref., referent.

^c^CI, confidence interval.

The results of the backward elimination logistic models for the entire study population are shown in Table 2, and subset analyses for each of the three socioeconomic groups are shown in Table 3. The tables include all the factors that remained in the backward elimination models. In Table 2, the logistic model for the entire population shows that drinking water from a faucet (unfiltered) or directly from a lake, river, or stream was associated with *T. gondii* infection, with ORs of 1.5- and 1.6-fold, respectively.

**Table 2:** Risk for *Toxoplasma gondii* seropositivity shown as odds ratios estimated with backward elimination logistic regression for all the three socioeconomic populations combined.^a^

		Wald confidence limits			
Variable	Odds ratio	Lower	Upper		p value
Age (yrs)					
0–9	Ref.^b^				
0–14	1.75	1.15		2.68	0.001^c^
15–19	2.84	1.75		4.63	<0.001^c^
20–29	4.36	2.52		7.55	<0.001^c^
30–39	8.84	4.30		18.20	<0.001^c^
40–49	10.81	5.18		22.56	<0.001^c^
50+	8.45	3.75		19.00	<0.001^cc^
Socioeconomic population					
Lower	Ref.				
Middle	0.48	0.32		0.72	<0.001^c^
Upper	0.14	0.08		0.24	<0.001^c^
Location					
Urban	Ref				
Suburban	1.56	1.07		2.28	0.022
Other Factors (ref. is absence of factor)					
Unfiltered water	1.23	0.97		1.74	0.081
Lake, river, or stream water	1.63	1.16		2.29	0.005^c^
Faucet water	1.54	1.17		2.03	0.002^c^
Kibbeh	0.69	0.50		0.96	0.027
Ice cream (in plastic pop-up bags)	1.32	1.01		1.73	0.046
Attend home shows	0.75	0.57		0.99	0.039
Eat in restaurants	0.69	0.52		0.92	0.011^c^
Region 1 (compared with other regions)	0.74	0.56		0.98	0.037

^a^All variables remaining in the model are shown.

^b^Ref., referent.

^c^Statistically significant (p<0.01, rounded).

**Table 3:** Risk for *T. gondii* seropositivity shown as odds ratios estimated with backward elimination logistic regression for each of the three socioeconomic populations^a^

		*Wald confidence limits*			
Variable	Odds ratio	Lower	Upper		p value
Lower socioeconomic group					
Age (yrs)					
0–9	Ref.				
15–19	4.31	1.15		16.13	0.300
20–29	9.78	2.10		45.46	0.036
30–39	10.02	2.13		47.20	0.036
40–49	5.83	1.60		21.28	0.076
50+	11.95	2.47		57.86	0.021
Location					
Urban	Ref.				
Rural	0.27	0.10		0.69	0.007^b^
Other factors (ref. is absence of factor)					
Unfiltered water	3.04	1.34		6.93	0.008^b^
Undercooked beef	2.89	1.05		7.96	0.040
Ate in restaurants	0.40	0.19		0.83	0.014^b^
Living in region 1	0.31	0.12		0.78	0.013^b^
Middle socioeconomic group					
Age (yrs)					
0–9	Ref.				
15–19	1.56	1.07		2.26	0.019
20–29	2.06	1.24		3.40	0.005^b^
30-39	4.70	2.10		10.50	<0.001^c^
40-49	4.62	2.03		10.50	<0.001^b^
50+	4.62	1.81		11.79	0.001^b^
Other factors (ref. is absence of factor)					
Female	0.74	0.54		1.01	0.061
Unfiltered water	1.67	1.22		2.30	0.002^b^
Lake river or stream water	1.80	1.17		2.76	0.008^b^
Kibbeh	0.64	0.44		0.95	0.026
Ice cream (in pop-up bags)	1.38	1.00		1.92	0.051
Attend home shows	0.72	0.52		0.98	0.037
Living in region 1	0.74	0.54		1.00	0.052
Lower socioeconomic group					
Age (yrs)					
0–9	Ref.				
40–49	18.24	4.36		76.30	<0.001^b^
50+	12.42	1.93		79.89	0.008^b^
Other factors (ref. is absence of factor)					
Female	0.50	0.26		0.99	0.046
Living in region 1	0.05	0.01		0.49	0.011^b^

^a^All variables remaining in the model are shown.

^b^Statistically significant (p<0.01, rounded) factors.

The logistic model for the lowest socioeconomic population (Table 3) shows that living in rural regions (OR 0.27, CI 0.10 to 0.69) as well as eating in restaurants (OR 0.40, CI 0.19 to 0.83) were protective for *T. gondii* infection; on the other hand, drinking unfiltered water increased the risk for *T. gondii* infection 3-fold (OR 3.04, CI 1.34 to 6.93). For the middle socioeconomic population, no variable was found to be protective for *T. gondii* infection, and as with the entire population, drinking unfiltered water or drinking directly from a lake, river, or stream increased the risk for *T. gondii* seropositivity 1.7- and 1.8-fold, respectively (Table 3). No risk factor other than age was associated with seropositivity for the upper socioeconomic population (Table 3), although living in region 1 of the city was protective in this group (OR 0.05, CI 0.01 to 0.49).

We then examined risk factors for the 360 persons with the strongest 25% of serologic reactions (412–1000 IU), compared with those who were seronegative (<12 IU). In addition to age and socioeconomic status, suburban vs. urban location (OR 2.8, 95% CI 1.8 to 4.2, p<0.001) and drinking unfiltered water (OR 2.0, 95% CI 1.4 to 2.8, p<0.001), or lake, river or stream water (OR=1.5, 95% CI 1.0 to 2.2, p=0.048), or faucet water (OR 1.7, 95% CI 1.2 to 2.5, p=0.001) vs. the absence of these water-related factors, increased the risk for high levels of *T. gondii* seropositivity. Eating in restaurants (OR 0.4, 95% CI 0.3 to 0.6, p<0.001) and living in region 1 compared with other regions (OR 0.4, 95% CI 0.3 to 0.6, p<0.001) decreased the risk for strongly reactive serologic tests.

We also determined risk factors for children 0–14 years of age; the strongest 25% of serologic reactions were compared to those with seronegative reactions in a logistic model (192 with strongest serologic reactions, 433 seronegative, total N=625). In this analysis, children belonging to the middle socioeconomic population had an increased risk for *T. gondii* seropositivity when they were compared with children in the lower soscioeconomic population (OR 2.0, 95% CI 1.3 to 3.1, p=0.002); those living in a suburban location had a higher risk than those in an urban location (OR 2.8, 95% CI 1.7 to 4.8, p<0.001); and those drinking unfiltered water had a higher risk (OR 2.5, 95% CI 1.6 to 3.9, p<0.001) than those who drank lake, river, or stream water (OR 1.9, 95% CI 1.1 to 3.3, p=0.020). Eating in restaurants (OR 0.4, 95% CI 0.3 to 0.6, p<0.001) and living in region 1 compared with living in other regions (OR 0.4, 95% CI 0.2 to 0.6, p<0.001) decreased the risk for *T. gondii* seropositivity.

## Discussion

The findings that age and socioeconomic status were the strongest predictors of *T. gondii* seropositivity in this study are consistent with those of other studies (*8*–*11*). However, to our knowledge, this study is the first to implicate the consumption of untreated or unfiltered water as a source of *T. gondii* infection in a disease-endemic area.

Most persons in the lower socioeconomic population complement their water supplies by constructing wells on their property. In this region of northern of Rio de Janeiro State, on average, water can be found only 9 m deep, and the well entrances are usually close to the ground. Contamination with oocysts from soil is likely to occur in floods or runoff that are frequent in Campos after rainfall. Since oocysts can survive long periods in water (*12*), the lower socioeconomic population frequently may be exposed to *T. gondii* oocysts from drinking water. In fact, most persons in the lower socioeconomic population were infected by the age of 15 (Figure).

Anecdotal reports imply that, in the past 15 years, the upper socioeconomic population in the city began to consume bottled spring water, and that, for the past 10 years, nearly 100% of the upper socioeconomic population utilizes this type of water for drinking and to rinse raw vegetables and fruits. This change is attributed to the poor taste of the municipal water, which has not been well accepted by the upper socioeconomic population.

Most *T. gondii* oocysts that contaminate water reservoirs and wells come from soil; however, in this study, contact with soil was not a risk factor for toxoplasmosis. In northern Rio de Janeiro State, water may be a more important means of ingesting oocysts. The lack of association between variables related to owning cats and seropositivity for toxoplasmosis in the logistic regression models is consistent with results of numerous other studies (*13*–*16*). Cats often spread oocysts away from their home, and feral cats may be responsible for much of the environmental contamination with oocysts. However, owning a cat was associated with *T. gondii* seropositivity in the univariate analysis and was more common among persons in the lower and middle socioeconomic groups than among the upper socioeconomic group.

Several other factors were associated with *T. gondii* seropositivity by univariate analysis, but not by multivariate analysis, for example, eating unpasteurized butter in the past 12 months. Unpasteurized butter could increase the risk for *T. gondii* infection if the butter was contaminated by oocysts from soil. However, unpasteurized butter was not one of the stronger risk factors, and the association with *T. gondii* seropositivity may be confounded by socioeconomic status, i.e., persons in lower socioeconomic groups (a strong risk factor for *T. gondii* seropositivity) may be more likely to eat unpasteurized butter. In fact, when we checked the data to determine the proportion of persons eating unpasteurized butter stratified by socioeconomic group, we found that persons in the lower socioeconomic group were more likely to report that they had eaten unpasteurized butter than were those in the middle or upper socioeconomic groups (39% vs. 32% and 24%, respectively). Consuming either unpasteurized cow’s milk or goat’s milk were not found to be risk factors. Drinking spring (bottled) water and attending home entertainment shows were associated with a decreased risk for *T. gondii* seropositivity by univariate analysis; these two factors were more common in the upper socioeconomic group than in the middle and lower socioeconomic groups.

In logistic regression, variables such as “living in region 1 of the city” and “eating in restaurants” were protective for the lower socioeconomic population. These findings may reflect differences in social conditions within the lower socioeconomic population. In fact, in region 1 the sanitary conditions of very poor communities are slightly better in comparison with those found in other regions of Campos. Another variable, “living in rural regions” (Table 3) was protective for the lower socioeconomic population. The closer proximity of animals (including cats) to human homes and the smaller space for deposition of animal waste in urban/suburban regions, thus increasing the possibility of water contamination with *T. gondii* ooccysts, might account for this difference within the lower socioeconomic population.

Our study is subject to a number of limitations. Because seropositivity for *T. gondii* persists for many years a direct temporal relationship between behaviors and *T. gondii* infection cannot be established. However, our findings were confirmed in subset analyses of persons who may have been infected in the more recent past, i.e., children and persons with high serologic titers.

Our study and those of others recently published on congenital toxoplasmosis in Brazil (*17*,*18*) implicate toxoplasmosis as an important health problem. Toxoplasmosis may be equally important in many other developing countries, where the lack of adequate sanitary conditions expose populations to a variety of diseases. Although some waterborne infections have been more thoroughly evaluated by the public health system, others, such as toxoplasmosis, remain to be investigated further to fully define the risk attributable to waterborne transmission.
